# Intestinal Parasite Community Dynamics in the Critically Endangered Chinese Alligator (*Alligator sinensis*): Multifactorial Insights From 18S rRNA Amplicon Sequencing

**DOI:** 10.1002/ece3.73706

**Published:** 2026-05-21

**Authors:** Mingxia Ge, Qingquan Chang, Qingyu Ge, Genjun Tu, Yongkang Zhou, Pingsi Yi, Hongji Sun, Jinhong Zhao

**Affiliations:** ^1^ Department of Medical Parasitology Wannan Medical College Wuhu China; ^2^ Anhui Provincial Key Laboratory of Conservation and Exploitation of Biological Resources, College of Life Sciences Anhui Normal University Wuhu China; ^3^ The National Nature Reserve of Chinese Alligator in Anhui Xuanzhou China

**Keywords:** 18S rRNA high‐throughput sequencing, Chinese alligators, conservation management, intestinal parasites

## Abstract

The intestinal parasite communities of vertebrates are shaped by complex interactions among biological, environmental, and anthropogenic factors. However, such dynamics remain poorly characterized in endangered the Chinese Alligator ( *Alligator sinensis* ). Here, we conducted a high‐throughput 18S rRNA amplicon survey of intestinal parasites in 149 fecal samples from the critically endangered Chinese alligators, spanning two geographic localities, four feeding regimens, three developmental stages, and 3 months. We identified 1524 eukaryotic parasite OTUs and 6606 nematode OTUs, revealing significant geographic variation in parasite composition, with higher richness in Wuhu than in Xuancheng populations. PERMANOVA further confirmed significant locality‐level differences in parasite community structure for both eukaryotic and nematode assemblages. Feeding regimen strongly influenced parasite diversity and community composition, with nematode communities exhibiting particularly pronounced responses, showing the lowest abundance and diversity in alligators maintained on a standardized catfish‐carp diet and highest in those fed diverse prey. Eukaryotic parasite richness declined markedly with host age, while nematode diversity showed pronounced depression in August. At finer taxonomic resolution, multiple parasite phyla and genera exhibited dynamic, factor‐specific responses to locality, diet, age, and sampling month, highlighting strong ecological filtering effects. Linear Discriminant Analysis Effect Size analysis uncovered specific parasite taxa associated with distinct factors. Our results firstly demonstrate the structure of parasite communities in *A. sinensis* , and clarified how they are shaped by interacting ecological and management factors, providing critical baseline data for future research.

## Graphical abstract

Our results firstly demonstrate the structure of parasite communities in *A. sinensis* and clarify how they are shaped by interacting ecological and management factors, providing critical baseline data for future research.

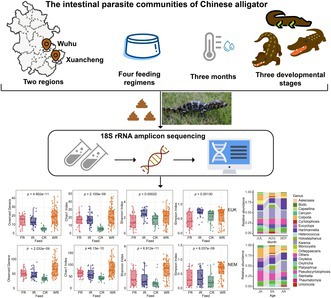

## 1 Background

The animal gastrointestinal tract hosts a diverse and complex array of ecosystems, which profoundly affect various aspects of host biology, including digestion, immune modulation, and overall health (Shortt et al. 2018). Among these pathogen communities, intestinal parasites play a critical role in shaping host health and survival, particularly in reptiles. Parasitic infections can significantly influence reptilian fitness, altering growth rates, reproductive success, and even mortality (Rinaldi et al. 2012; Bower et al. 2019; Gafoor and Tardieu 2025). Factors such as diet, host physiology, and environmental conditions have been widely reported to affect intestinal parasite prevalence (Jejaw et al. 2014; Pavlova et al. 2018; Chalabi 2024; Mo et al. 2024; Zhang et al. 2025). Additionally, certain parasites pose a threat of zoonotic transmission to those who interact closely with animals, including handlers, caretakers, and owners (Mendoza‐Roldan et al. 2020; Varela et al. 2022).

The Chinese alligator ( *Alligator sinensis* ), a highly threatened species endemic to China, is one of the most ancient crocodilians still extant today. This species, which has survived since the Mesozoic era, once populated the large wetlands, marshes, and ponds along the lower reaches of the Yangtze River, currently listed as “critically endangered” on the International Union for Conservation of Nature and Natural Resources (IUCN) Red List (Thorbjarnarson et al. 2002; Bosi et al. 2022). Currently, fewer than 100 mature wild individuals remain, distributed in five narrow localities in Anhui Province (Yang et al. 2023). Despite conservation efforts since 1979, including the establishment of breeding farms, the wild population remains at an extremely high risk of extinction (Thorbjarnarson et al. 2002; Thorbjarnarson and Xiaoming 2009; Watanabe 2009). Consequently, research on the pathogen biology of the Chinese alligator holds significant and far‐reaching implications for the conservation of this imperiled species (Sun, Chen, et al. 2025; Sun, Li, et al. 2025).

In zoological research, non‐invasive sampling methods, such as fecal collection, are crucial for studying endangered species, as they minimize stress and avoid direct handling (Yu et al. 2024). However, despite the growing body of research on the gut microbiome of reptiles (Kohl et al. 2016; Jiang et al. 2017; Tang et al. 2019; Willson et al. 2019; Zhou et al. 2020), there is a relative paucity of studies focusing on the intestinal parasitic community in the Chinese alligator. Existing reports are mostly confined to the morphological identification of parasites, which often target limited parasite taxa and are constrained by lower resolution and discriminatory power compared to molecular approaches (Zhao et al. 2014, 2016). In contrast, high‐throughput sequencing, as an emerging molecular diagnostic approach, provides notable advantages in cost‐effectiveness, sequencing depth, and accuracy, and has been successfully applied to investigations of gut parasites (Zeng et al. 2022; Wu et al. 2025), providing a more comprehensive and deeper understanding of microbial communities and parasitic infections. Nevertheless, the application of this technology to the detection of intestinal parasites in the Chinese alligator remains a critical knowledge gap that needs to be addressed.

In the present study, we utilized 18S rRNA profiling to systematically investigate the intestinal parasite community in the Chinese alligator. We comprehensively analyzed the relationships between parasitic prevalence and key factors, including feeding practices, geographic distribution, climate variations, and developmental stages of the alligators. This research aimed to provide novel insights into the parasitic ecology of this endangered species, offering critical baseline data for future conservation strategies.

## 2 Methods

### 2.1 Sample Collection and DNA Extraction

The collection of the flesh fecal samples was carried out once per month on the 15th of July, August, and September 2024. Sampling was conducted in the morning with the assistance of the animal caretakers and was performed on the same dates across all enclosures and both localities. After collection, each sample was carefully sorted and individually labeled to avoid cross‐contamination and mix‐ups. Fresh fecal samples were collected in sterile bags and promptly stored in an ice box container. All samples were transported to the laboratory within 2 h and stored at −80°C until DNA extraction.

A total of 149 fecal samples from the captive Chinese alligator ( *Alligator sinensis* ) were collected across two primary geographic localities in Anhui Province: Wuhu and Xuancheng (Table 1). The Xuancheng samples were further categorized according to three distinct breeding zones: free‐range rearing group (FR, *n* = 28), intensive rearing group (IR, *n* = 29), and common rearing group (CR, *n* = 20). The Wuhu samples were designated as the Wuhu rearing group (WR). Specifically, the WR group primarily consumed fish, bovine liver, chicken byproducts (heads/liver), and rabbit; the FR group's diet consisted mainly of large fish, rabbit, and river snails; the CR group was fed predominantly catfish and carp, whereas the IR group received small miscellaneous fish, crucian carp, and chopped large fish pieces. Additionally, the Xuancheng specimens were stratified by age into three developmental stages: juvenile alligator (JA group, < 4 years, *n* = 16), subadult alligator (SA group, 4–6 years, *n* = 13), and adult alligator (AA group, > 7 years, *n* = 48). The age grouping was conducted based on the growth, development, and reproductive patterns of the Chinese alligator (Wang RP et al. 2006). To account for temporal variation, samples were collected from two localities across 3 months: July (JUL group, *n* = 51), August (AUG group; eukaryotic amplification *n* = 47, nematode amplification *n* = 44), and September (SEP group, *n* = 51). Detailed information on individual samples and their group assignments is provided in Table S1.

**TABLE 1:** Grouping strategy and sequencing details of samples in this study.

Library primer	Sampling location		Months			Age			Feeding regimens				Average reads	
			July (JUL)	August (AUG)	September (SEP)	Juveniles (JA, < 4 years)	Subadults (SA, 4–6 years)	Adults (AA, > 7 years)	Free‐range rearing (FR)	Intensive rearing (IR)	Common rearing (CR)	Wuhu rearing (WR)	Raw	Clean
EUK	Wuhu	72	24	23	25	0	0	72	0	0	0	72	86,795	86,442
	Xuancheng	77	27	24	26	16	13	48	28	29	20	0	85,258	84,814
NEM	Wuhu	69	24	20	25	0	0	69	0	0	0	69	115,858	114,395
	Xuancheng	77	27	24	26	16	13	48	28	29	20	0	113,291	112,264

*Note:* Universal eukaryotic primers (EUK); Nematode‐specific primers (NEM).

Total DNA was extracted from fecal samples using the DNA extraction kit (MOBIO, CA, USA) following the manufacturer's guidelines, and the purity and concentration of DNA were detected using Nanodrop One (Thermo Fisher Scientific, MA, USA). The purified DNA was used for polymerase chain reaction (PCR), restriction digestion, and next‐generation sequencing. Extracted DNA integrity was tested using 1.0% agarose gel electrophoresis.

### 2.2 PCR Amplification and DNA Sequencing

To enhance the detection spectrum of parasitic organisms in fecal samples, two distinct primer pairs were designed to amplify the 18S ribosomal gene for broad eukaryotic detection and specific nematode identification. Each sample underwent high‐throughput sequencing of 18S rDNA using two pairs of primers. Three samples failed to amplify against the nematode‐specific primer region, thus a total of 146 DNA samples were conducted to amplification against nematode‐specific primer regions. The primer sequences are detailed as follows: Universal eukaryotic primers (hereafter referred to as ‘EUK’ primers) (Ju et al. 2025): 528F forward: 5′‐GCGGTAATTCCAGCTCCAA‐3′, 706R reverse: 5′‐AATCCRAGAATTTCACCTCT‐3′; Nematode‐specific primers (hereafter referred to as ‘NEM’ primers) (Bongiorno et al. 2019): 3NDf forward: 5′‐GGCAAGTCTGGGTGCCAG‐3′, 1132rmod reverse: 5′‐TCCGTCAATTYCTTTAAGT‐3′. The PCR amplification was performed in 50 μL reactions containing: 50 ng template DNA, 1 μL of each primer (10 μM), 25 μL of 2× Premix Taq (EX Taq Version 2.0 with dye; Takara Bio, Dalian, China), and nuclease‐free water to adjust the final volume. The PCR protocol was as follows: an initial denaturation phase at 94°C for 5 min, succeeded by 30 cycles of a three‐step process comprising 94°C for 30 s, 52°C for 30 s, and 72°C for 30 s, concluding with a final extension at 72°C for 10 min.

Subsequently, the concentration of the PCR products was evaluated using GeneTools software (Version 4.03.05.0, SynGene). Based on the principle of equal mass, the volume of each sample was determined and the PCR products were combined. The mixed PCR products were purified utilizing a gel recovery kit, with the target DNA fragments eluted using TE buffer. The library construction adhered to the standardized procedure outlined in the ALFA‐SEQ DNA Library Prep Kit. The size of the library fragments was assessed on a Qsep400 High‐Throughput Nucleic Acid‐Protein Analysis System, and the library concentration was measured employing Qubit 4.0 software (Thermo Fisher Scientific, Waltham, USA). The constructed amplicon libraries are subjected to PE250 sequencing on the Illumina platform (Guangdong Magigene Biotechnology Co. Ltd., Guangzhou, China).

### 2.3 Sequencing Data Processing

The low‐quality reads and primers were removed using fastp software (Chen et al. 2018) (an ultra‐fast all‐in‐one FASTQ preprocessor, version 0.14.1, “https://github.com/OpenGene/fastp”) and cutadapt software (Martin 2011) (“https://github.com/marcelm/cutadapt/”), thus obtaining quality‐controlled paired‐end Clean Reads. Total numbers of reads obtained for each sequencing library are shown in Table S2. All the clean reads from all samples were clustered into Operational Taxonomic Units (OTUs) using Uparse software (Edgar 2013) with default parameters. Representative sequences from each OTU were then taxonomically annotated at the species level by aligning them against the SILVA database (v138) (Quast et al. 2013) using usearch‐sintax/blast, with a confidence threshold set to 0.8. Taxonomic classification was assigned across seven hierarchical levels: kingdom, phylum, class, order, family, genus, and species. Given the primary objective of this study was to assess the diversity of intestinal parasites in the Chinese alligator, we excluded OTUs from the EUK primers library that did not belong to the following taxonomic groups (Yu et al. 2024): Tubulinea, Apicomplexa, Apusozoa, Cercozoa, Choanozoa, Ciliophora, Foraminifera, Loukozoa, Metamonada, Heterolobosea, Radiolaria, Platyhelminthes, Euglenozoa, Nematoda, Arthropoda, and Acanthocephala. This filtering step ensured that subsequent analyses focused exclusively on parasite‐related taxa.

### 2.4 Statistical Analysis

To comprehensively explore the factors influencing intestinal parasite community in Chinese alligators, we delved into the diversity of parasites under varying factor groupings. Alpha diversity and PERMANOVA analyses were conducted using the R package “vegan” (version 2.7‐3) (Oksanen et al. 2026), with all OTUs analyzed at the genus level. Alpha diversity indices, including Observed genera richness, Chao1, Shannon, and Simpson indices, often deviate from normality and homoscedasticity assumptions. Therefore, to ensure robust statistical inference, non‐parametric factorial analyses were conducted using the Aligned Rank Transform (ART) approach as implemented in the “ARTool” R package (Elkin et al. 2021). A Bray–Curtis dissimilarity matrix, based on the relative abundance of OTUs, was computed to perform permutational multivariate analysis of variance (PERMANOVA). The top five most abundant phyla and 20 most abundant genera of EUK‐targeted and NEM‐targeted parasites were evaluated by examining variations across different grouping factors. The significance of differences in relative abundance across groups at different taxonomic levels was assessed using the kruskal.test function in the R platform. In analyses of parasite diversity and community structure, each focal factor (e.g., month, age, or feeding mode) was evaluated while controlling for other relevant variables as covariates to account for potential confounding effects.

For biomarker discovery, we performed linear discriminant effect size analysis (LEfSe) via the “microeco” R package (Liu et al. 2021) to identify parasite taxa significantly associated with different groups. Statistical significance in the analysis was determined based on LDA scores exceeding 3.0 and an α‐level of 0.05. The visualization process in this study was mainly realized using the “ggplot2” (Wickham et al. 2024), “ggpubr” (Kassambara and Kassambara 2024), “ggalluvial” (Brunson and Read 2024), and “patchwork” (Pedersen 2024) packages in the R platform.

## 3 Results

### 3.1 Study Overview

**FIGURE 1:**
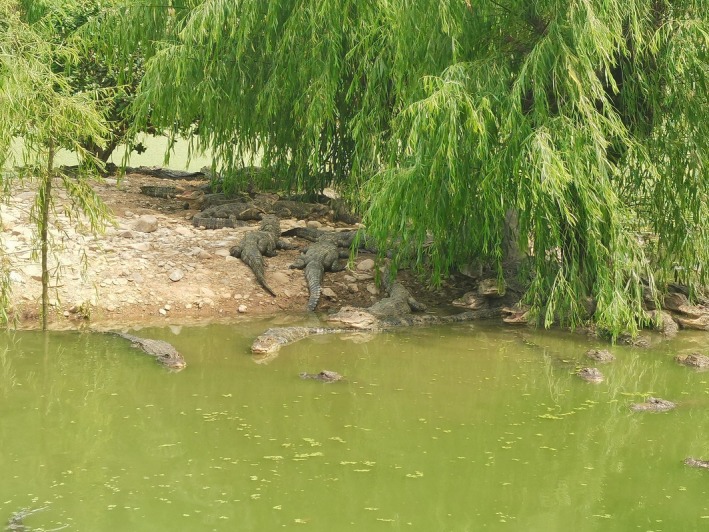
Photograph of captive adult Chinese alligators, taken at the National Nature Reserve of Chinese Alligator in Xuanzhou, Anhui Province, by the corresponding author Jinhong Zhao.

The interactions between hosts and parasites are often modulated by a wide array of factors, spanning biological, environmental, and anthropogenic drivers (McNew et al. 2021; Gsell et al. 2023). In this study, we collected 149 fecal samples from Chinese alligators (Figure 1), stratified by geographic location, feeding practice, developmental age, and collection date (Table 1 and Table S1). After quality filtering and chimera removal, a total of 16,537,639 high‐quality reads (NEM primers; mean ± SD: 113,272 ± 21,292) and 12,754,576 high‐quality reads (EUK primers; mean ± SD: 85,601 ± 6654) were retained, accounting for 98.9% and 99.5% of the raw sequences, respectively (Table S2). Amplification with universal eukaryotic primers (EUK primers) identified 1524 OTUs, classified as parasitic taxa including Tubulinea, Apicomplexa, Cercozoa, Ciliophora, Heterolobosea, Platyhelminthes, Euglenozoa, Nematoda, and Arthropoda. Additionally, nematode‐specific primers (NEM primers) yielded 6606 OTUs, providing enhanced resolution for nematode diversity analysis (Tables S3 and S4).

### 3.2 Prevalence and Diversity of Parasites in Fecal Samples of Chinese Alligators

Parasites load was prevalent in fecal samples from Chinese alligators across both two localities in this study. EUK primer analysis revealed 23 parasite classes common to both Wuhu and Xuancheng populations, while Chilopoda, Monogenea, and Diplopoda were exclusively detected in Xuancheng (Figure S1A). At the order level, we identified eight taxa in NEM primer. Among these, Rhabditida, Trichinellida, Dorylaimida, Monhysterida, Strongylida, and Plectida were found in both localities; Chromadorida was exclusively observed in the Wuhu locality, and Mermithida was only detected in the Xuancheng locality (Figure S1B). These findings demonstrate significant geographical variation in the intestinal parasite composition of Chinese alligators.

**FIGURE 2:**
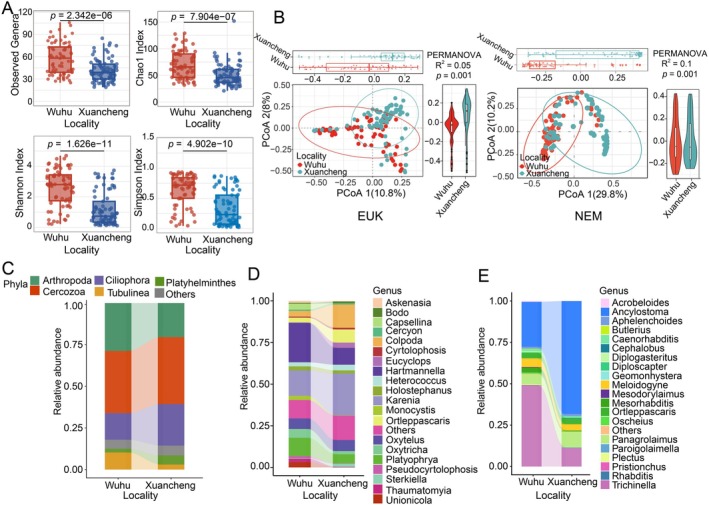
Comparative analysis of intestinal parasite diversity and abundance in Chinese alligators across localities. (A) Alpha diversity comparison of nematode parasites at the genus level (NEM primer) between localities. Statistical significance was assessed by Aligned Rank Transformation (ART) ANOVA. (B) Principal coordinate analysis (PCoA) of eukaryotic parasites (left) and nematodes (right) based on Bray‐Curtis distances of amplicon sequence variant (ASV) relative abundance. Ellipses denote 95% confidence intervals for each locality. (C–E) Locality‐level variations in parasite community composition across taxonomic levels. Alluvial plots display relative abundance patterns of the five most abundant phyla (C), and the 20 most abundant genera (D, E). Data shown represent amplification results from (D) EUK primers and (E) NEM primers.

To further explore differences among localities in gut parasite diversity, we first assessed the alpha diversity of the parasite communities. The results demonstrated that Wuhu area exhibited significantly higher gut parasite community richness (Observed‐species, Chao1) than Xuancheng area for both EUK and NEM primer sets (EUK: Observed genera, *F* = 23.88, *p* = 2.679e‐06; Chao1, *F* = 20.05, *p* = 1.519e‐05; NEM: Observed genera, *F* = 24.23, *p* = 2.342e‐06; Chao1, *F* = 26.7, *p* = 7.904e‐07). Notably, community diversity (Shannon, Simpson) exhibited statistically significant spatial variation among sampling localities solely in NEM‐specific analyses (Shannon: *F* = 53.68, *p* = 1.626e‐11; Simpson: *F* = 44.68, *p* = 4.902e‐10), whereas EUK‐based comparisons failed to reach statistical significance (Figure 2A and Figure S1C). Principal coordinate analysis (PCoA) using Bray–Curtis distance at the OTU level failed to uncover pronounced differences in either EUK‐targeted or NEM‐targeted parasite community structure across different localities. However, PERMANOVA analysis demonstrated statistically significant compositional differences (for EUK: *F* = 2.6, *R* ^2^ = 0.05, *p* = 0.001; for NEM: *F* = 5.5, *R* ^2^ = 0.1, *p* = 0.001; 999 permutations, Bray–Curtis distance) (Figure 2B).

Subsequently, we explored the dynamic changes in the community composition at different taxa levels (phylum, genus). As outlined in the taxonomic annotations of the 1524 OTUs (Table S3), Cercozoa emerged as the most abundant phylum among EUK‐targeted parasites in both localities, followed by Arthropoda, Ciliophora, Tubulinea, and Platyhelminthes (Figure 2C). However, notable shifts in relative abundance were observed between localities. Specifically, Tubulinea was significantly less abundant in Xuancheng than in Wuhu (*W* = 12.71, *p* = 0.00036), while Platyhelminthes was significantly more abundant in Xuancheng (*W* = 10.07, *p* = 0.0015). At the genus level, the EUK‐targeted parasites showed a significant rise in the relative abundance of *Karenia* (*W* = 4.78, *p* = 0.029) and *Eucyclops* (*W* = 5.42, *p* = 0.02) in the Xuancheng locality, whereas *Hartmannella* (*W* = 6.22, *p* = 0.013), *Platyophrya* (*W* = 12.86, *p* = 0.00033), *Oxytricha* (*W* = 6.21, *p* = 0.013), *Unionicola* (*W* = 22.99, *p* = 1.63e‐06), and *Capsellina* (*W* = 39.24, *p* = 3.75e‐10) declined (Figure 2D). For NEM‐targeted parasites, the relative abundance of *Ancylostoma* (*W* = 42.88, *p* = 5.83e‐11) showed a significant rise in the Xuancheng locality, accompanied by a decreasing trend in the relative abundance of *Trichinella* (*W* = 58.93, *p* = 1.63e‐14) (Figure 2E).

### 3.3 Variations in Gut Parasite Abundance and Diversity of Chinese Alligators Across Feeding Regimens, Age and Climates

**FIGURE 3:**
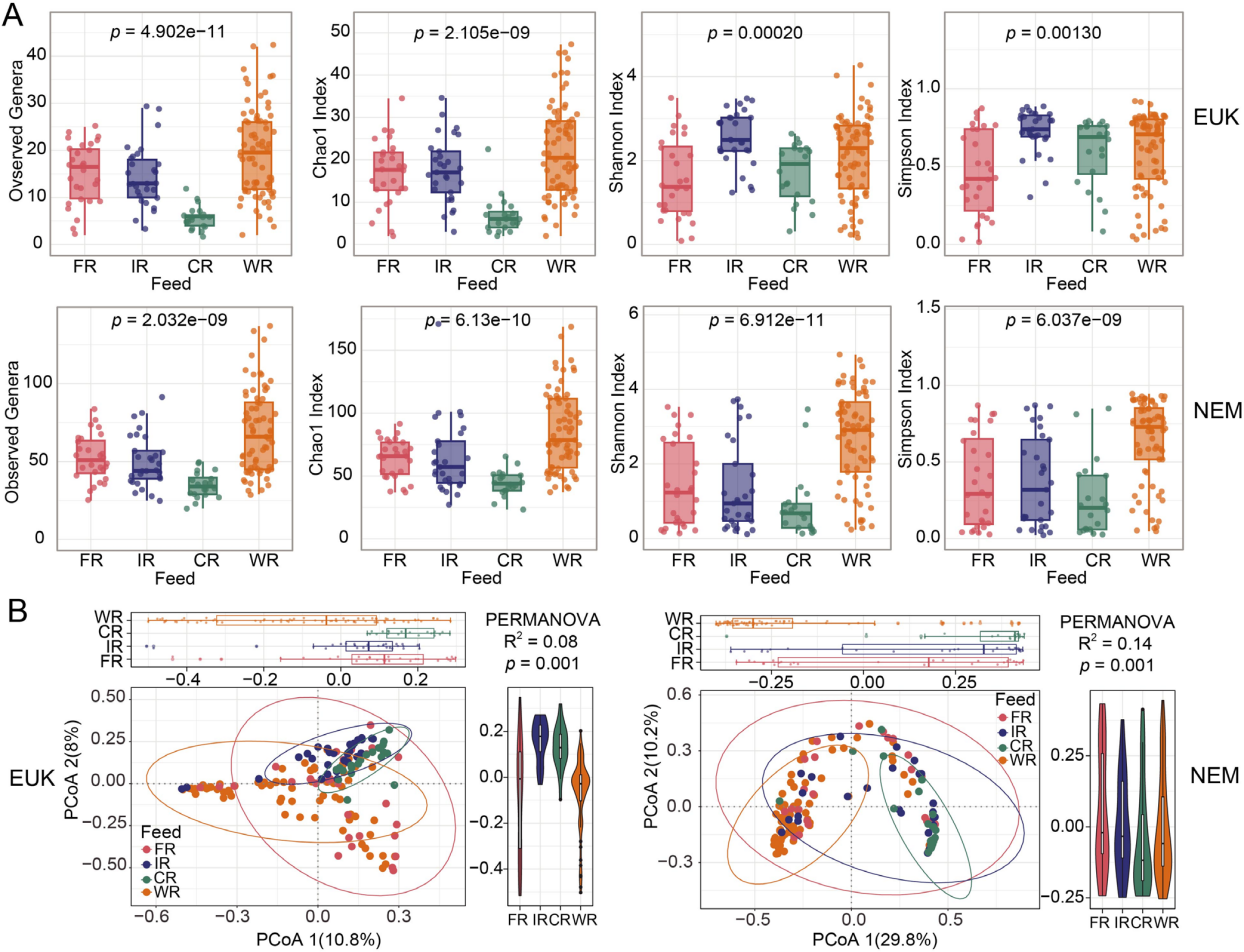
Impact of dietary regimen on intestinal parasite diversity in Chinese alligators. (A) Alpha‐diversity metrics of eukaryotic parasites (top row) and nematodes (bottom row) across four feeding strategies. Box‐and‐whisker plots display observed genera, Chao1 index, Shannon index, and Simpson's evenness. Statistical significance was evaluated by Aligned Rank Transformation (ART) ANOVA. (B) Beta‐diversity assessment via principal coordinate analysis (PCoA) of Bray–Curtis dissimilarities computed from ASV relative abundances. Confidence ellipses (95%) illustrate clustering by feeding regimen. Left panels: Eukaryotic parasites (universal eukaryotic primers); right panels: Nematode parasites (nematode‐specific primers).

Our analysis of both α‐diversity and β‐diversity unveiled that Chinese alligators under different feeding regimens exhibited significant differences in the richness and community structure of intestinal parasites, with these differences being evident for both EUK‐targeted (eukaryotic parasites) and NEM‐targeted (nematode) parasite groups (Figure 3A,B). Notably, nematode communities (amplified with NEM primers) displayed significantly lower richness and diversity in the CR feeding group compared to other regimens, with the highest values observed in the WR group, followed by the FR group (Figure 3A). Similarly, eukaryotic parasites (EUK primer) exhibited minimal richness in the CR group, while the FR group showed the lowest diversity indices. PERMANOVA analysis confirmed significant differences in parasite community structure across all four feeding regimens for both parasite types (for EUK: *F* = 3.253, *R* ^2^ = 0.08, *p* = 0.001; for NEM: *F* = 5.948, *R* ^2^ = 0.14, *p* = 0.001; 999 permutations, Bray–Curtis distance; Figure 3B).

**FIGURE 4:**
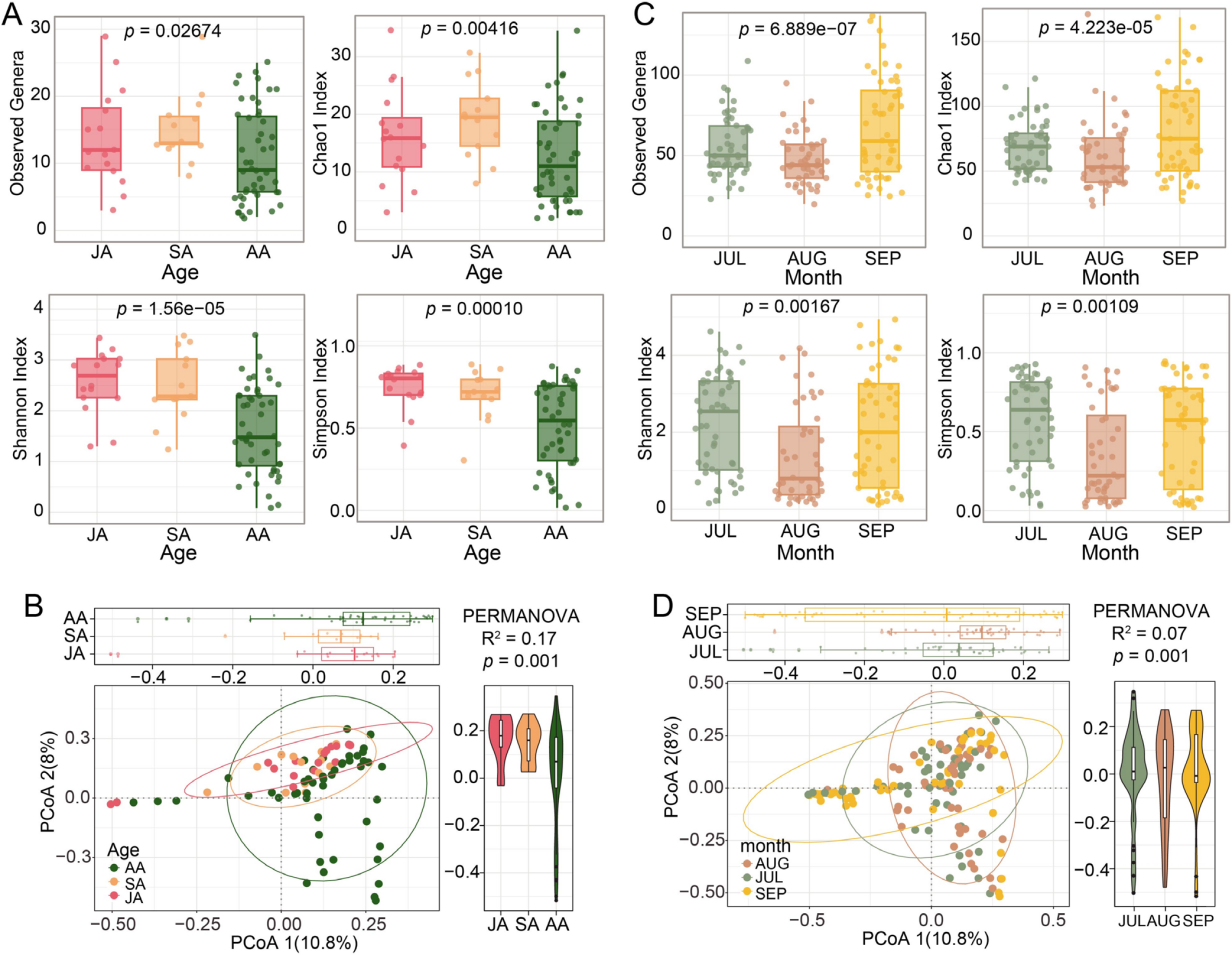
Drivers of intestinal parasite diversity in Chinese alligators. (A, B) Eukaryotic parasite (EUK) diversity across developmental stages: (A) Alpha diversity comparison among age groups. (B) Beta diversity visualized by principal‐coordinate analysis (PCoA) of Bray–Curtis dissimilarities among ASV profiles; 95% confidence ellipses are shown. (C, D) Sampling months variation in parasite diversity: (C) Nematode alpha indices across months. (D) Eukaryotic parasite β‐diversity colored by collection month.

We next examined age‐related and climate variations in parasite diversity. The eukaryotic parasites (EUK primer) richness and diversity were found significantly reduced in adult alligators (AA, > 7 years) compared to both juveniles (JA, < 4 years) and subadults (SA, 4–6 years) (Figure 4A). Community structure analysis further revealed distinct eukaryotic parasite assemblages among age groups (*F* = 2.91, *R* ^2^ = 0.17, *p* = 0.001; 999 permutations, Bray–Curtis distance; Figure 4B). However, no significant differences in richness, diversity, or community structure of nematode parasites (NEM primer) were found across age groups (Figure S2A,B). Additionally, sampling month analysis identified August as the period with significantly depressed nematode richness and diversity (Observed genera: *F* = 15.78, *p* = 6.889e‐07; Chao1: *F* = 10.86, *p* = 4.223e‐05; Shannon: *F* = 6.71, *p* = 0.02; Simpson: *F* = 7.18, *p* = 0.001) compared to July and September collections (Figure 4C), though community structure remained stable across climate changes (Figure S2C). Eukaryotic parasites exhibited variations across sampling dates. in both community structure (*F* = 2.154, *R* ^2^ = 0.07, *p* = 0.001; 999 permutations, Bray–Curtis distance) and richness, while maintaining consistent diversity measures (Figure 4D, Figure S2D).

### 3.4 Parasite Community Composition Is Dynamically Influenced by Multiple Factors

**FIGURE 5:**
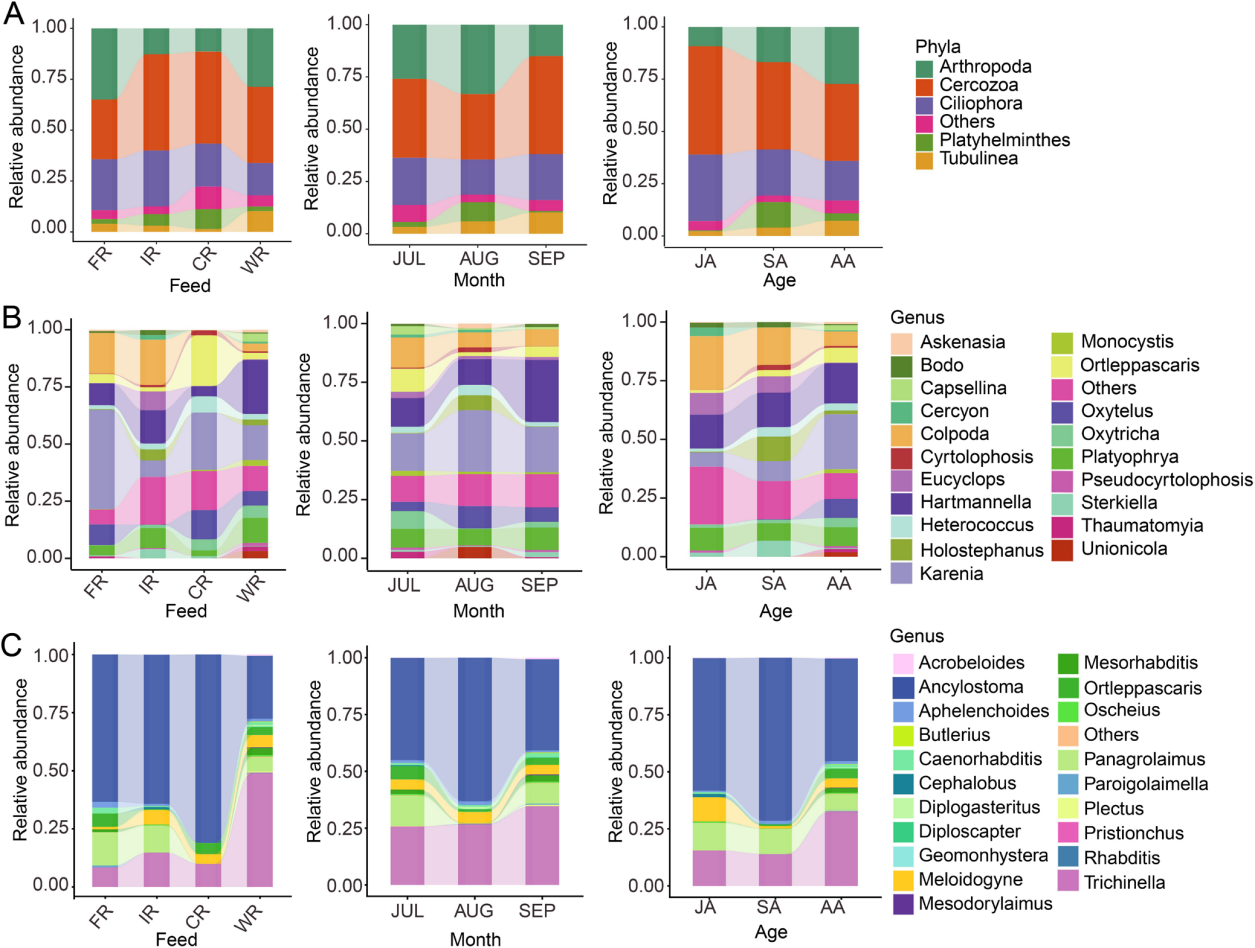
Taxonomic composition of intestinal parasites across feeding practices, collection months, and developmental stages in Chinese alligators. (A) Relative abundance of the five most abundant eukaryotic parasite phyla across different rearing practices, collection months, and age groups. Color‐coded phyla are indicated in the legend (right). Stacked bars represent proportional composition for each sample group. (B) Composition of the 20 most abundant eukaryotic parasite genera, and (C) the 20 most abundant nematode genera, analyzed under the same experimental conditions as in (A).

The relative abundance at different taxonomic levels revealed dynamic variations of EUK‐ and NEM‐targeted parasites in Chinese alligator feces under varying feeding regimens, host age, and climate factors (Figure 5). Analysis of the top five eukaryotic parasites at the phylum level revealed significant variations in relative abundance across different dietary regimens. Cercozoa exhibited a significantly higher relative abundance in the IR and CR groups (*W* = 7.85, *p* = 0.049). Comparative analysis between localities revealed distinct parasitic profiles: Tubulinea showed higher relative abundance in Wuhu‐fed alligators compared to the three feeding regimes in Xuancheng (*W* = 16.31, *p* = 0.00098), whereas Ciliophora abundance was conversely diminished. At the genus level, *Karenia* displayed particularly high abundance in FR specimens but was notably scarce in IR samples (*W* = 14.23, *p* = 0.0026). Similarly, *Oxytelus* showed reduced prevalence in IR specimens (*W* = 30.21, *p* = 1.25e‐06), while *Hartmannella* (*W* = 18.87, *p* = 0.00003) exhibited pronounced increases in CR specimens. Among NEM‐targeted parasite species, *Ancylostoma* reached peak abundance in CR samples with minimal detection in WR group (*W* = 45.39, *p* = 7.63e‐10), contrasting with *Trichinella* which predominated in WR samples (*W* = 60.24, *p* = 5.23e‐13). A particularly striking finding was the near absence of *Panagrolaimus* in CR fecal samples, despite its relatively high abundance in FR group (*W* = 25.31, *p* = 1.33e‐05).

In addition to the aforementioned findings, we observed differential variations in the relative abundance of intestinal parasites in Chinese alligators at the phylum and genus levels across different sampling dates and developmental stages (Figure 5). Specifically, in terms of sampling date variations, the relative abundance of Cercozoa (*W* = 10.06, *p* = 0.0065) was lowest in August compared to July and September. Conversely, Arthropoda (*W* = 17.87, *p* = 0.00013) and Platyhelminthes (*W* = 16.35, *p* = 0.00028) exhibited peak abundance in August. At the genus level, *Hartmannella* (*W* = 6.22, *p* = 0.047) showed elevated relative abundances in September, whereas *Karenia* (*W* = 8.44, *p* = 0.015) and *Ancylostoma* (*W* = 9.41, *p* = 0.0091) were most prevalent in August. From a developmental perspective, the abundances of Arthropoda exhibited an increasing trend with host maturation (*W* = 8.34, *p* = 0.016), while Cercozoa and Ciliophora gradually declined (*W* = 7.46, *p* = 0.024 and *W* = 8.65, *p* = 0.031, respectively). Of particular note, Platyhelminthes achieved significantly higher relative abundance in the SA group relative to the other two age groups (*W* = 7.36, *p* = 0.025). Genus‐level analysis revealed elevated abundance of *Oxytelus*, *Ortleppascaris*, and *Trichinella* in adult alligators (*W* = 13.69, *W* = 16.61, *W* = 7.01; *p* = 0.0011, 0.00025, and 0.03, respectively), in contrast to notable reductions in *Colpoda*, *Eucyclops*, *Ancylostoma*, and *Panagrolaimus* (*W* = 37.23, *W* = 39.78, *W* = 6.35, W = 12.40; *p* = 8.22e‐09, 2.3e‐09, 0.042, and 0.002, respectively) (Figure 5).

### 3.5 Strong Associations Between Certain Parasite Taxa and Specific Factors

Subsequent LEfSe analysis revealed several parasite clades exhibiting statistically significant differences among sample groups. In the EUK set, *Levicoleps biwae* (Colepidae), *Platyophrya* (Cyrtolophosidida: Platyophryidae), *Oxytricha longigranulosa* (Sporadotrichida: Oxytrichidae), *Monocystis* (Eugregarinorida: Monocystidae), and *Capsellina* (Rhogostomidae) showed significant differential abundance between localities (LDA score > 3, *p* < 0.05; Figure S3A). Within the NEM set, *Ancylostoma* (Strongylida: Ancylostomatidae) was significantly richer in Xuancheng locality, whereas *Trichinella* (Trichinellida: Trichinellidae), Longidoroidea, and *Mesorhabditis* (Rhabditida: Rhabditidae) exhibited specificity to Wuhu locality (Figure S3B).

**FIGURE 6:**
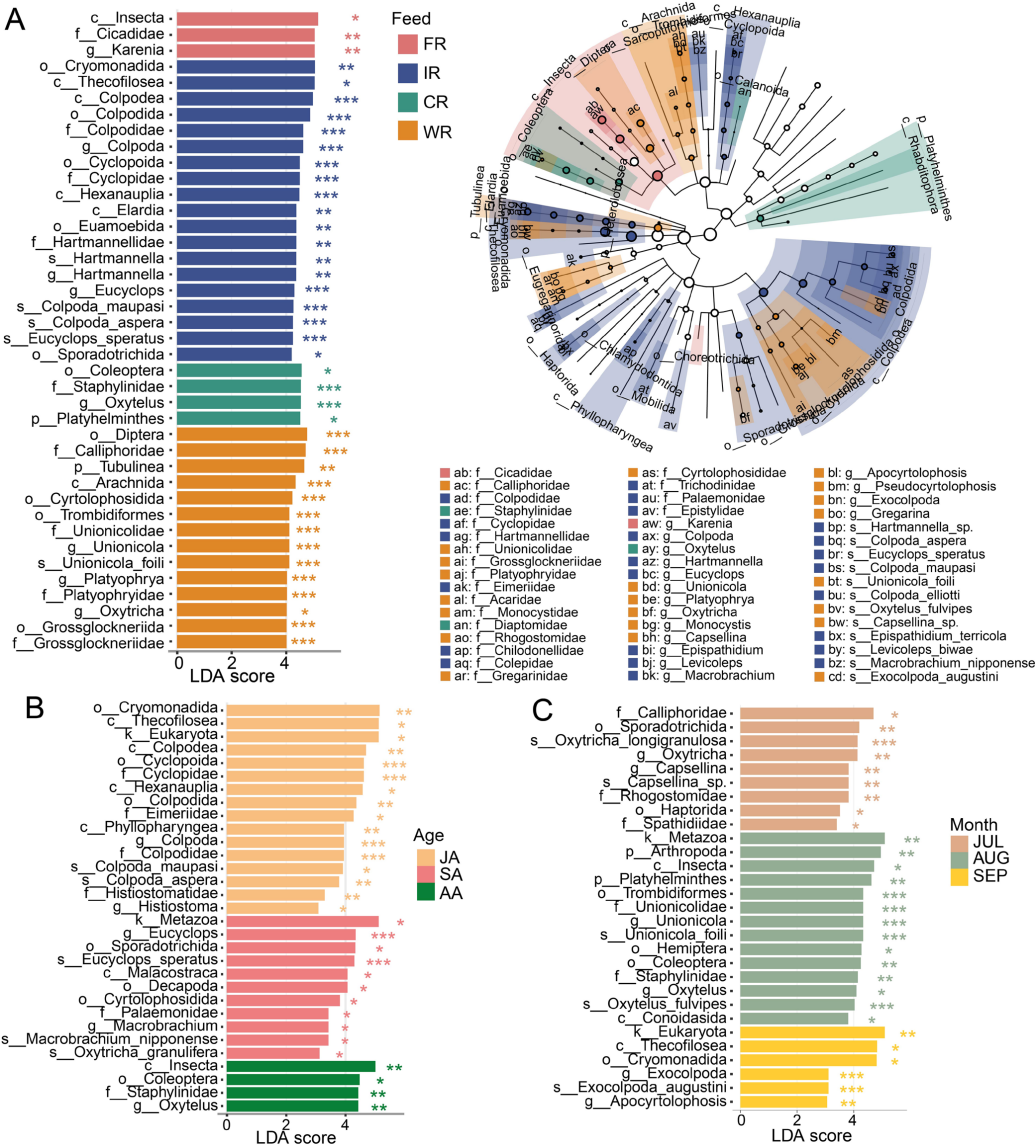
Indicator eukaryotic parasites in the gut of Chinese alligators under distinct effect factors. Linear discriminant analysis effect size (LEfSe) was applied to detect taxa whose relative abundances differed significantly among experimental factors (*p* < 0.05). Bars display the logarithmic LDA scores (> 3.0) of the most discriminant taxa; extended cladograms illustrate their phylogenetic placement. (A) Influential taxa associated with feeding regimens. (B) Taxa distinguishing developmental stages. (C) Taxa characteristic of sampling months.

Analysis of Chinese alligator fecal samples under different feeding regimes revealed distinct parasitic signatures. Based on EUK data, several taxa within Cercozoa, Tubulinea, and Ciliophora were identified as characteristic of IR feeding mode, including Cryomonadida, *Hartmannella*, *Colpoda maupasi* , and *Colpoda aspera* (Figure 6A). Conversely, CR mode showed significant enrichment of *Ortleppascaris* sp. (Rhabditida: Heterocheilidae) and *Ancylostoma* (Strongyloidea: Ancylostomatidae) (Figure S4A). Notably, Cryomonadida, *Colpoda maupasi* and *Colpoda_aspera* were also prominently enriched in alligators of JA group, along with significant representation of Eimeriidae and Phyllopharyngea (Figure 6B). Distinct enrichment patterns emerged in other groups: Sporadotrichida and *Oxytricha granulifera* predominated in SA samples, while *Ortleppascaris gigantea* was characteristic of AA specimens (Figure 5B and Figure S4B). Furthermore, we identified sampling month variations in parasitic biomarkers within Chinese alligator intestines. July samples demonstrated preferential enrichment of Sporadotrichida, *Oxytricha longigranulosa*, *Capsellina* sp., and *Panagrolaimidae*, whereas September specimens showed significant abundance of Platyhelminthes, Conoidasida, *Exocolpoda augustini*, *Apocyrtolophosis* and Rhabditidae (Figure 5C and Figure S4C).

## 4 Discussion

The present study provides a comprehensive analysis of the intestinal parasite community in the captive Chinese alligator ( *Alligator sinensis* ), an endangered species endemic to China. Our findings reveal a complex interplay of factors influencing the prevalence and diversity of the parasitic community, including geographical location, feeding regimens, host developmental stage, and climate variations. These insights are critical for developing effective conservation strategies, as intestinal parasites are an integral part of the host's ecosystem, influencing immune modulation, nutrient cycling, and overall physiological homeostasis.

Our findings reveal that distinct feeding regimens in the Chinese alligator serve as a pivotal determinant of parasitic community patterns within the intestinal tract. The CR feeding group exhibited the lowest nematode diversity and abundance (Figure 3), likely attributable to their controlled, monotonous diet, which limits exposure to potential parasite vectors (Prati et al. 2020). By contrast, the FR and WR feeding groups were fed a more diverse and varied diet, and as a result, showed higher parasite richness. Previous studies have established a strong correlation between host dietary behavior and endoparasite diversity, with evidence demonstrating that hosts with broader dietary spectra exhibit significantly higher parasite richness (Thieltges and Poulin 2016; Guerrero‐Sanchez et al. 2023). Intriguingly, the surprisingly low parasite diversity observed in the FR group implies potential influences from additional ecological determinants, including the nutritional quality of food resources and host population density. Nutritional deficiencies have been linked to immunosuppression, thereby increasing vulnerability to parasitic infections and promoting heavier parasite burdens (Ezenwa 2004; Knapp et al. 2013). The relationship between diet standardization and parasite burden underscores the importance of controlled feeding protocols in captive programs.

In most vertebrate systems, juveniles are more susceptible than adults because their adaptive immune system is still maturing (Ashby and Bruns 2018; Buckingham et al. 2023). Host ontogeny is also a key determinant of parasite community structure (Izhar and Ben‐Ami 2015; Hallinger et al. 2018; Guardone et al. 2024), our analysis of intestinal parasites in the Chinese alligator revealed that adult individuals harbor significantly lower eukaryotic parasite diversity and abundance than both juveniles and subadults (Figure 4A,B). Specifically, the reduction of Eimeriidae, *Colpoda*, *Eucyclops*, *Ancylostoma* and *Panagrolaimus* in adult alligators may reflect either immune system maturation or behavioral adaptations that limit parasite exposure. This observed pattern corresponds with previous findings from intestinal parasite surveys in Chinese alligators (Zhao et al. 2014). Furthermore, the increased abundance of *Oxytelus*, *Ortleppascaris* and *Trichinella* in adult alligators may indicate that these parasites are relatively insensitive to age‐associated immune maturation in the host, or alternatively, that they benefit from ecological shifts within the aging gut environment.

The critically endangered Chinese alligator, whose remaining wild populations are primarily confined to the lower Yangtze River basin in Anhui Province (Pan et al. 2019), faces heightened vulnerability to climate change impact (Yang et al. 2024; Sun, Chen, et al. 2025; Sun, Li, et al. 2025). Our results reveal that climate variations have a significant impact on gastrointestinal parasite community in captive Chinese alligators. Specifically, compared to July and September, both the diversity and abundance of gastrointestinal parasites, particularly nematodes, declined significantly in August (Figure 4C,D and Figure S2C,D). This phenomenon may be attributed to the transition into a hot and dry period in the Wuhu and Xuancheng localities during August (Zhou et al. 2021). High temperatures and arid conditions likely exert suppressive effects on the eggs and larvae of certain parasites (Beveridge et al. 1989; Gamboa 2005; Mignatti et al. 2016), while reduced water availability may diminish vector populations, subsequently lowering the likelihood of parasite establishment. Notably, the study sites of Wuhu and Xuancheng selected in this research are located within the natural distribution range of wild Chinese alligators in China. Furthermore, captive individuals are raised in outdoor breeding facilities. The captive alligators in this study are exposed to identical climatic and geographical conditions as wild populations, with highly analogous dietary resources. Such similarities consequently foster comparable compositions of intestinal parasite communities. Accordingly, the gastrointestinal parasite community observed in our captive population can, to a certain extent, serve as a proxy for that of wild populations. This finding also provides important implications for the conservation and targeted management of wild Chinese alligator populations.

In conclusion, this study advances our understanding of parasitic ecology in the captive Chinese alligator, demonstrating that parasite communities are shaped by a complex interplay of biotic and abiotic factors, such as developmental stages of alligators, types of food, management methods of feeding, as well as seasonal variations like temperature and humidity, etc. Therefore, our findings underscore the importance of implementing comprehensive prevention and control strategies to ensure the health of Chinese alligators in captivity and prevent parasitic infections during the management of breeding: regularly and systematically remove feces from the shoreline to prevent accumulation and subsequent contamination of the external environment; ensure that food sources such as fish, chickens, and rabbits are single‐origin and have undergone regular deworming during their breeding process, to effectively reduce the probability of parasitic infections; enhance the crocodiles' resistance and overall health through the addition of nutritional supplements to support their immune function; regular deworming is still an effective prevention and control strategy during the breeding process; additionally, strengthen the health training of breeders to ensure they master scientific hygiene management methods, conduct environmental hygiene monitoring, and promptly identify and address potential health risks. However, the analysis of intestinal parasite community in this study did not definitively distinguish whether these parasites were endemic to the Chinese alligator or that entered the digestive tract incidentally through food intake. These strategies should take into account developmental stages, climate variations, locality‐level characteristics, and feeding practices. Future research should incorporate metagenomic and immunological approaches to further elucidate the mechanistic links between parasite dynamics and host health. Particularly, systematic studies on the life cycles of parasites are necessary to clarify the relationship with the Chinese alligator whether these parasites are endemic to the Chinese alligator or not. Additionally, future studies should also focus on collecting fecal samples from wild Chinese alligators to analyze their intestinal parasite communities, thereby laying a theoretical foundation for the prevention and control of parasitic diseases and the long‐term conservation of this endangered species.

## Author Contributions

**Mingxia Ge:** conceptualization (lead), data curation (lead), investigation (lead), methodology (lead), software (lead), writing – original draft (equal), writing – review and editing (equal). **Qingquan Chang:** formal analysis (equal), investigation (equal), methodology (equal), writing – original draft (equal). **Qingyu Ge:** investigation (supporting), methodology (supporting). **Genjun Tu:** investigation (supporting), methodology (supporting). **Yongkang Zhou:** investigation (supporting), methodology (supporting). **Pingsi Yi:** investigation (supporting), methodology (supporting). **Hongji Sun:** conceptualization (equal), formal analysis (equal), investigation (equal), resources (equal), writing – review and editing (equal). **Jinhong Zhao:** conceptualization (lead), funding acquisition (equal), supervision (lead), validation (lead), visualization (lead), writing – review and editing (supporting).

## Funding

This work was supported by the Key Projects of Natural Science Research in Universities of Anhui Province, 2025AHGXZK31066; the Key Program in the Youth Elite Support Plan in Universities of Anhui Province, gxyqZD2016171; the Doctoral Research Start‐up Fund of Wannan Medical College, WYRCQD2023019; School and Enterprise Cooperation Project, H202536.

## Conflicts of Interest

The authors declare no conflicts of interest.

## Supporting information


**Figure S1:** Comparative analysis of intestinal parasite diversity in Chinese alligators across different localities. (A‐B) Venn diagrams illustrating the shared and unique intestinal parasite taxa between the two localities at different taxonomic levels. (A) Eukaryotic primer amplification results (left to right: class, family, and genus levels); (B) Nematode‐specific primer amplification results (left to right: order, family, and genus levels). (C) Alpha diversity analysis of intestinal parasites amplified by eukaryotic primers in Chinese alligators from the two localities. Statistical significance was determined using Aligned Rank Transformation (ART) ANOVA.
**Figure S2:** Diversity of parasites in fecal samples of Chinese alligators across developmental stages and sampling months. (A) Alpha diversity (left) and beta diversity (right) of nematode parasites different ages. (B) Beta diversity of nematode parasites among different months. (C) Alpha diversity of eukaryotic parasites among distinct months.
**Figure S3:** Indicator parasites in the gut of Chinese alligators in different geographic localities. (A) EUK primer set; (B) NEM primer set.
**Figure S4:** Indicator intestinal nematode parasites of Chinese alligators under distinct effect factors. (A) Influential taxa associated with feeding regimens. (B) Taxa distinguishing developmental ages. (C) Taxa characteristic of sampling months.

**Table S1:** Grouping information of samples in this study.

**Table S2:** Sequencing quality metrics for the two primer pairs.

**Table S3:** Filtered OTU table used for downstream analyses in this study.

**Table S4:** Summary table with the parasite classes identified for each locality, feeding regimen, age and month group.

## Data Availability

The raw sequence data reported in this study have been deposited in NCBI Sequence Read Archive under accession number PRJNA1318566.
