# Supplementary material for: Intestinal Parasite Community Dynamics in the Critically Endangered Chinese Alligator (Alligator sinensis): Multifactorial Insights From 18S rRNA Amplicon Sequencing
Source: Ecol Evol. 2026 May 21;16(5):e73706. doi: 10.1002/ece3.73706 (PMC13240499; doi:10.1002/ece3.73706)
Supplement: Supplementary file 1 — Figure S1: Comparative analysis of intestinal parasite diversity in Chinese alligators across different localities. (A‐B) Venn diagrams illustrating the shared and unique intestinal parasite taxa between the two localities at different taxonomic levels. (A) Eukaryotic primer amplification results (left to right: class, family, and genus levels); (B) Nematode‐specific primer amplification results (left to right: order, family, and genus levels). (C) Alpha diversity analysis of intestinal parasites amplified by eukaryotic primers in Chinese alligators from the two localities. Statistical significance was determined using Aligned Rank Transformation (ART) ANOVA. Figure S2: Diversity of parasites in fecal samples of Chinese alligators across developmental stages and sampling months. (A) Alpha diversity (left) and beta diversity (right) of nematode parasites different ages. (B) Beta diversity of nematode parasites among different months. (C) Alpha diversity of eukaryotic parasites among distinct months. Figure S3: Indicator parasites in the gut of Chinese alligators in different geographic localities. (A) EUK primer set; (B) NEM primer set. Figure S4: Indicator intestinal nematode parasites of Chinese alligators under distinct effect factors. (A) Influential taxa associated with feeding regimens. (B) Taxa distinguishing developmental ages. (C) Taxa characteristic of sampling months. [file ECE3-16-e73706-s005.docx]

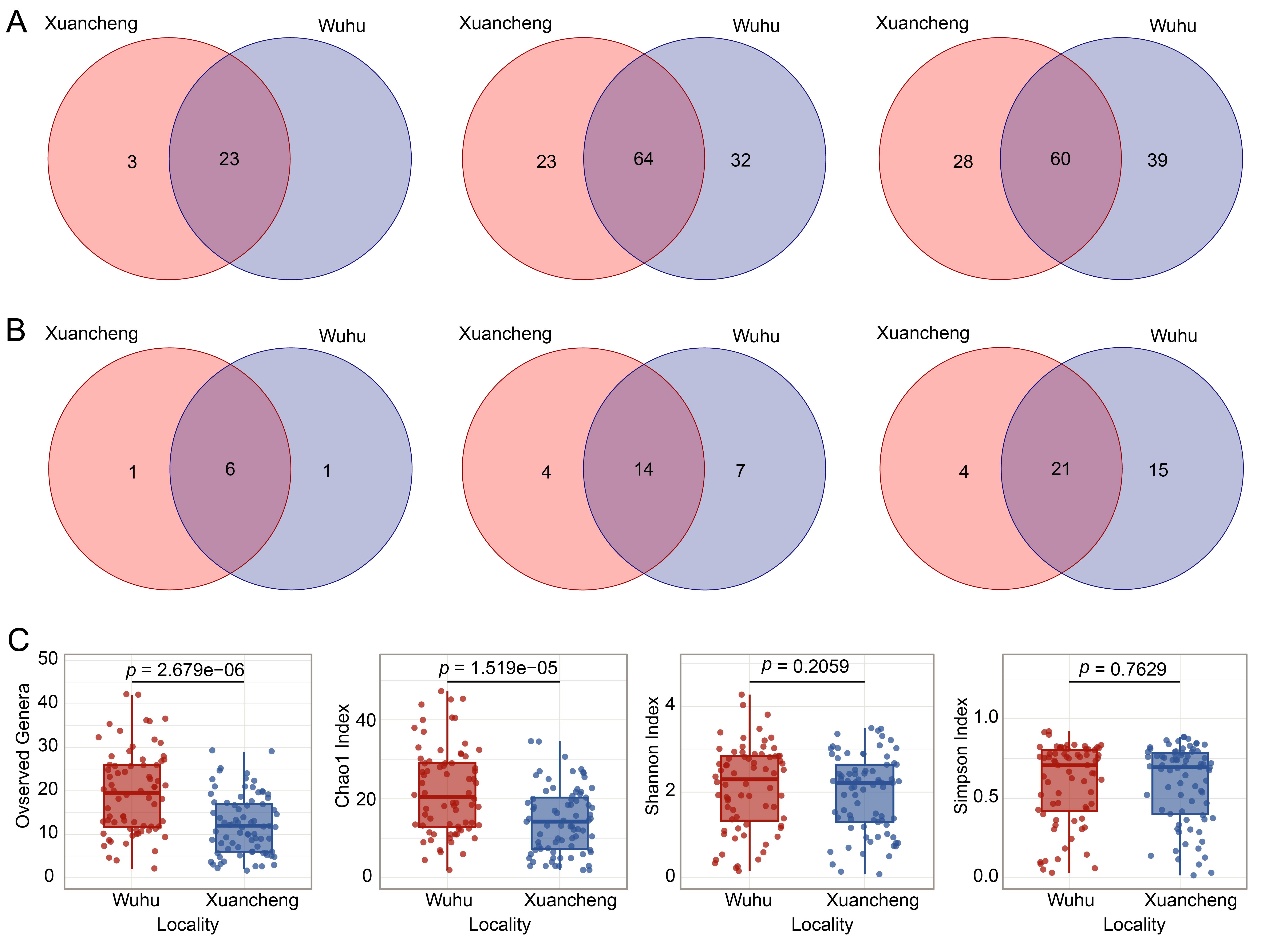


Figure S1. Comparative analysis of intestinal parasite diversity in Chinese alligators across different localities. (A-B) Venn diagrams illustrating the shared and unique intestinal parasite taxa between the two localities at different taxonomic levels. (A) Eukaryotic primer amplification results (left to right: class, family, and genus levels); (B) Nematode-specific primer amplification results (left to right: order, family, and genus levels). (C) Alpha diversity analysis of intestinal parasites amplified by eukaryotic primers in Chinese alligators from the two localities. Statistical significance was determined using Aligned Rank Transformation (ART) ANOVA.


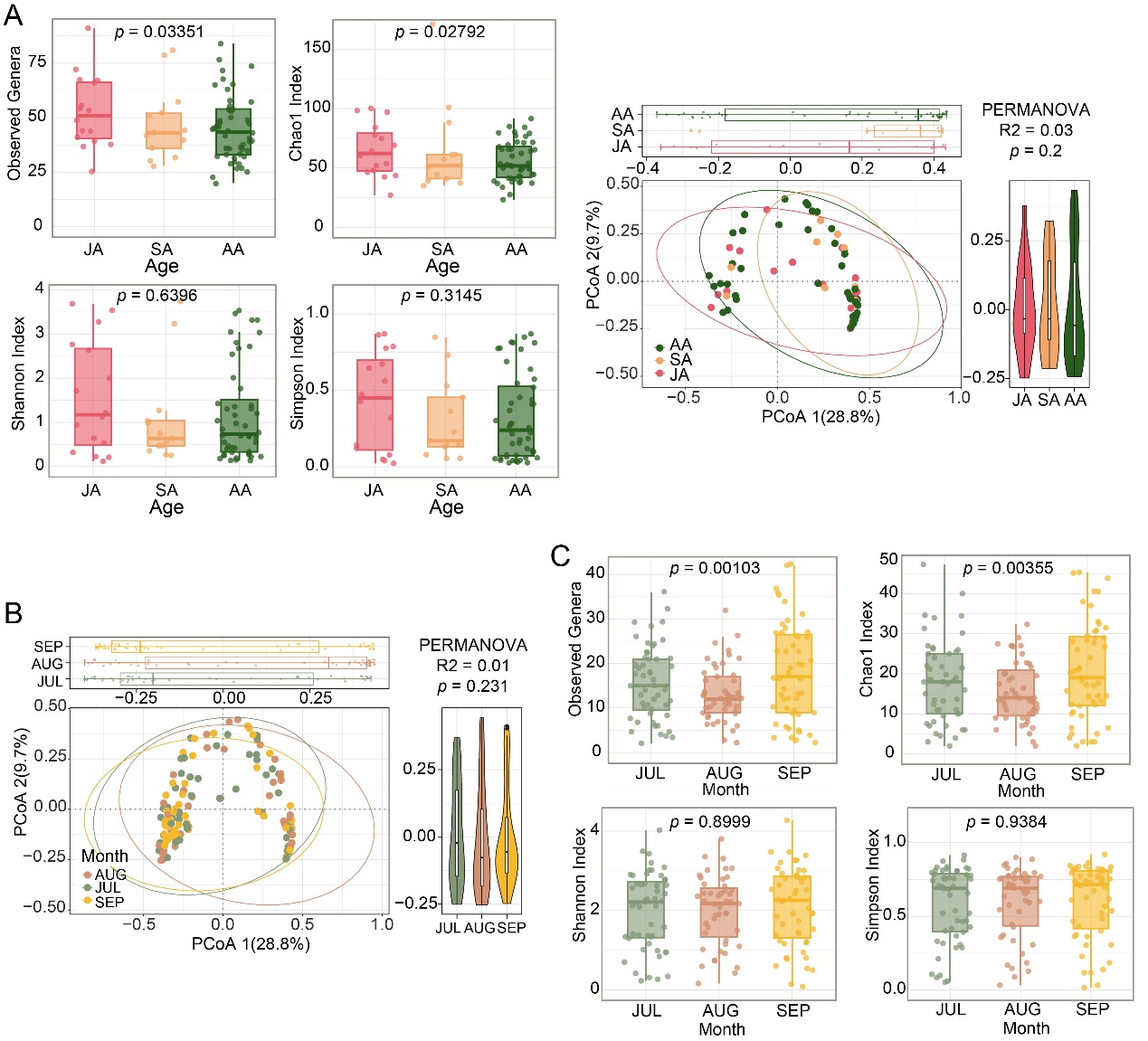


Figure S2. Diversity of parasites in fecal samples of Chinese alligators across developmental stages and sampling months. (A) Alpha diversity (left) and beta diversity (right) of nematode parasites different ages. (B) Beta diversity of nematode parasites among different months. (C) Alpha diversity of eukaryotic parasites among distinct months.


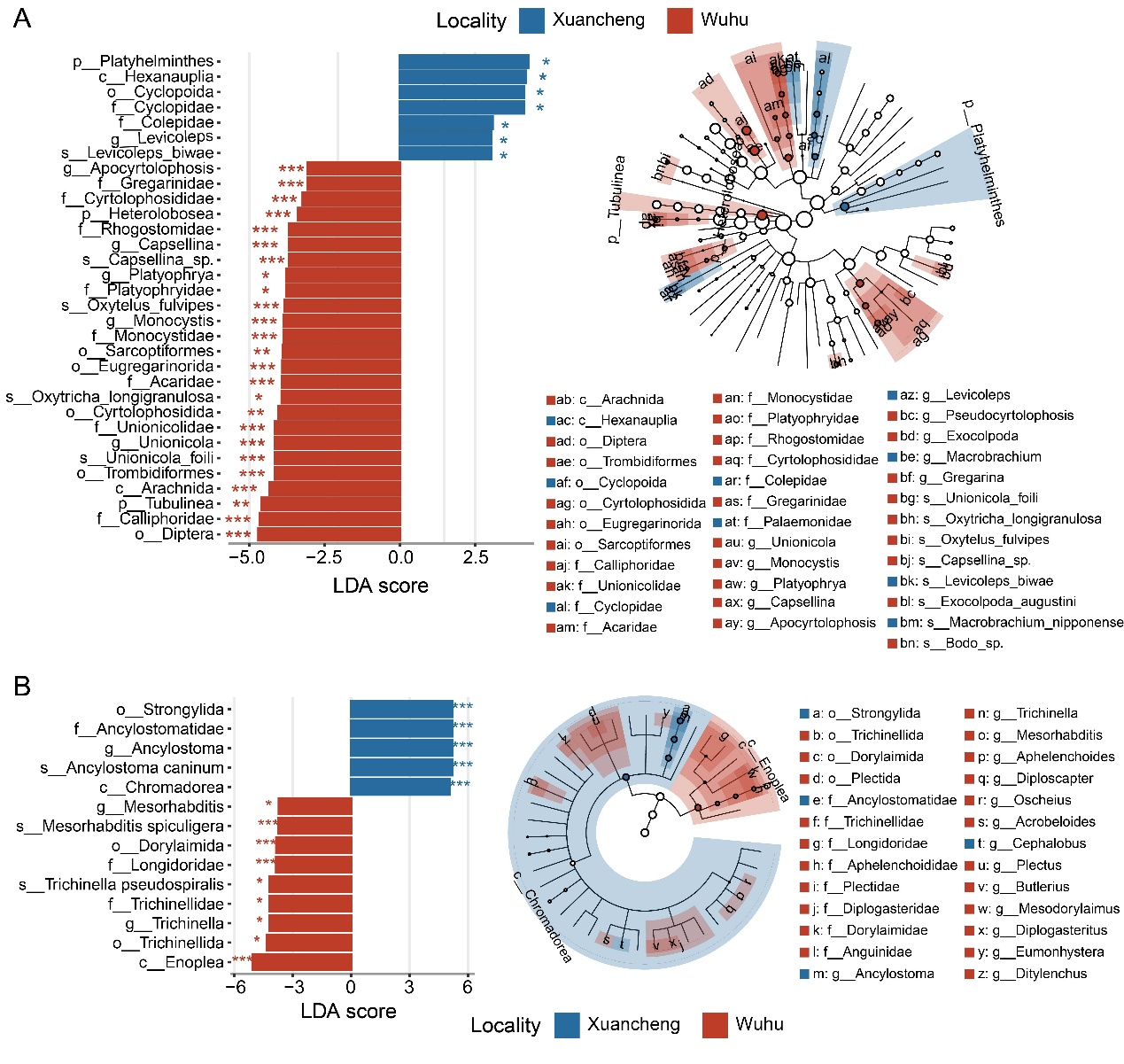


Figure S3. Indicator parasites in the gut of Chinese alligators in different geographic localities. (A) EUK primer set; (B) NEM primer set.


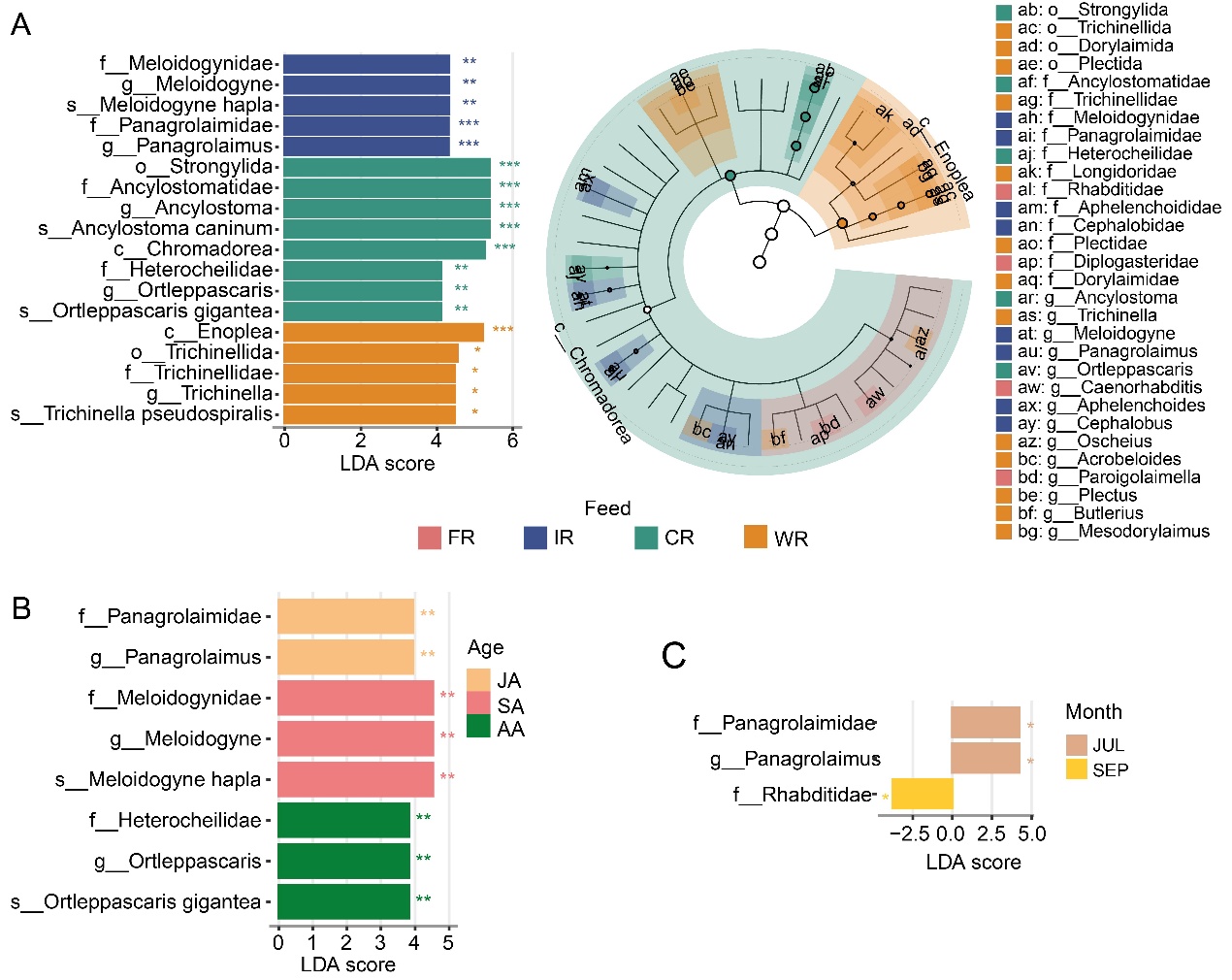


Figure S4. Indicator intestinal nematode parasites of Chinese alligators under distinct effect factors. (A) Influential taxa associated with feeding regimens. (B) Taxa distinguishing developmental ages. (C) Taxa characteristic of sampling months.

Table 1. Grouping strategy and sequencing details of samples in this study.

Table S1. Grouping information of samples in this study.

Table S2. Sequencing quality metrics for the two primer pairs.

Table S3. Filtered OTU table used for downstream analyses in this study.

Table S4. Summary table with the parasite classes identified for each locality, feeding regimen, age and month group.
